# The landscape of vaccines in China: history, classification, supply, and price

**DOI:** 10.1186/s12879-018-3422-0

**Published:** 2018-10-04

**Authors:** Yaming Zheng, Lance Rodewald, Juan Yang, Ying Qin, Mingfan Pang, Luzhao Feng, Hongjie Yu

**Affiliations:** 1 Key Laboratory of Surveillance and Early-warning on Infectious Disease, Division of Infectious Disease, Chinese Center for Disease Control and Prevention, Beijing, China; 2 World Health Organization, Beijing, China; 3 School of Public Health, Key Laboratory of Public Health Safety, Ministry of Education, Fudan University, Shanghai, China

**Keywords:** Vaccine, History, Classification, Supply, Price, China

## Abstract

**Background:**

Vaccine regulation in China meets World Health Organization standards, but China’s vaccine industry and immunization program have some characteristics that differ from other countries. We described the history, classification, supply and prices of vaccines available and used in China, compared with high-and middle-incomes countries to illustrate the development of Chinese vaccine industry and immunization program.

**Methods:**

Immunization policy documents were obtained from the State Council and the National Health and Family Planning Commission (NHFPC). Numbers of doses of vaccines released in China were obtained from the Biologicals Lot Release Program of the National Institutes for Food and Drug Control (NIFDC). Vaccine prices were obtained from Chinese Central Government Procurement (CCGP). International data were collected from US CDC, Public Health England, European CDC, WHO, and UNICEF.

**Results:**

Between 2007 and 2015, the annual supply of vaccines in China ranged between 666 million and 1,190 million doses, with most doses produced domestically. The government’s Expanded Program on Immunization (EPI) prevents 12 vaccine preventable diseases (VPD) through routine immunization. China produces vaccines that are in common use globally; however, the number of routinely-prevented diseases is fewer than in high- and middle-income countries. Contract prices for program (EPI) vaccines ranged from 0.1 to 5.7 US dollars per dose - similar to UNICEF prices. Contract prices for private-market vaccines ranged from 2.4 to 102.9 US dollars per dose - often higher than prices for comparable US, European, and UNICEF vaccines.

**Conclusion:**

China is a well-regulated producer of vaccines, but some vaccines that are important globally are not included in China’s EPI system in China. Sustained and coordinated effort will be required to bring Chinese vaccine industry and EPI into an era of global leadership.

**Electronic supplementary material:**

The online version of this article (10.1186/s12879-018-3422-0) contains supplementary material, which is available to authorized users.

## Background

Approximately 700 million vaccine doses are produced annually in China, making China one of the world’s largest producers of vaccines [1, 2]. China’s National Regulatory system for vaccines passed assessments by the World Health Organization (WHO) in 2011 and 2014, indicating that China’s vaccine regulatory oversight meets WHO/international standards [1]. Vaccines are made available through the government’s Expanded Program on Immunization (EPI) at no charge for all children up to 14 years of age [3]. These government-purchased vaccines are called Category 1 vaccines under the Regulations on the Administration of Vaccine and Vaccination. In contrast, private-sector (Category 2) vaccines, such as Haemophilus influenzae type b vaccine (Hib), rabies vaccine, and influenza vaccine (InfV), are available in China, but are usually paid for out-of-pocket, as they are included in neither the EPI system nor government health insurance.

China’s vaccine industry and immunization program have some differences compared with other countries’ industries and programs, but an overview of the Chinese vaccine industry and immunization effort has not been published in the international scientific literature. In order to allow international peers know better about the development of Chinese vaccine industry and immunization program and share experience, we analyzed selected aspects of vaccines and immunization in China and report the history, immunization policies, classification, supply, and price of vaccines in comparison with selected high- and middle-income countries.

## Methods

### Vaccine supplied, expanded program of immunization and ***categorization***

We obtained names of vaccines supplied in China between 1930s and 2016 from Applied Vaccination Handbook, the Biologicals Lot Release program of the National Institutes for Food and Drug Control (NIFDC), China Food and Drug Administration (CFDA) (http://www.nicpbp.org.cn/CL0001/). We determined official classification of vaccines into Category 1 or 2, and, when appropriate, year of integration into EPI from the documents, “Expanded National Immunization Program Planning and Implementation” by the National Health and Facility Planning Commission (NHFPC) (http://www.nhfpc.gov.cn) and “Applied Vaccination Handbook” [4]. We obtained year of licensure from CFDA (http://samr.cfda.gov.cn/WS01/CL0001/) and from the document, “Applied Vaccination Handbook” [3]. We obtained immunization schedules from the Chinese Center for Disease Control and Prevention (China CDC) [5] and “Applied Vaccination Handbook” [4].

We compared the list of vaccine of China with high-income countries including UK, US and middle-income countries including Brazil, Russia, India and South Africa. We obtained lists of vaccines available in the United States (US) and the United Kingdom (UK) from US CDC [6] and Public Health England [7]. Lists of vaccines and diseases prevented through routine immunization for China, US, UK, Brazil, Russia, India, and South Africa were obtained from China CDC, UNICEF, and WHO [8].

### Vaccine supply

We obtained the numbers of doses of vaccines released in China between 2007 and 2015 from NIFDC. Officially-classified Category 1 vaccines were: oral poliovirus vaccine (OPV), Group A meningococcal polysaccharide vaccine (MenA), Group A and C meningococcal polysaccharide vaccine (MenAC), hepatitis A live attenuated (HepA-L), hepatitis B vaccine (HepB), Bacillus Calmette-Guerin vaccine (BCG), diphtheria-tetanus-pertussis whole cell vaccine (DTwP), diphtheria-tetanus-pertussis acellular vaccine (DTaP), diphtheria and tetanus combined vaccine (DT), tetanus toxoid vaccine (TT), live attenuated Japanese encephalitis vaccine (JEV-L), inactivated Japanese encephalitis vaccine (JEV-I), measles-rubella vaccine (MR), measles, mumps combined vaccine (MM), measles-mumps-rubella vaccine (MMR), live attenuated measles vaccine (MV-L), mumps vaccine (Mumps), rubella vaccine (Rubella), hemorrhagic fever with renal syndrome (HFRS), leptospirosis vaccine (Leptospira), and anthrax vaccines (Anthrax). Pandemic H1N1 vaccine (H1N1) was classified as Category 1 because it was purchased by the government and made available at no cost to those vaccinated [9]. All other vaccines were Category 2, private-sector vaccines.

### Prices

We obtained vaccine prices from the Chinese Central Government Procurement (CCGP) office and from vaccine contracts published in the E-procured system by departments of health or CDCs in central, provincial, autonomous regions, municipal, city, and county-level governments [10]. We used “NIP vaccine” and “Category 2 vaccine” as separate keywords to search for vaccine prices. For analyses, we used the lowest price for each vaccine, consistent with procurement practices. We compared procurement prices in China with those of US, Europe and low-income countries. Vaccine prices of US were obtained from US CDC Vaccine Price List [6]. Vaccine prices for European countries were collected from “Review of Vaccine Price Data, 2013” by WHO European [11]. A total of 24 European countries reported their procurement prices for 41 vaccines. The median price for each vaccine were used in the comparison. Many low-income countries can obtain vaccines at low prices from UNICEF; we therefore used vaccine price data published by UNICEF instead of that of low- income countries [12]. The exchange rate for US dollars vs Chinese Yuan was 6.2. Cost data were collected between 2013 and 2015.

## Results

### Vaccine list

**Fig. 1:**
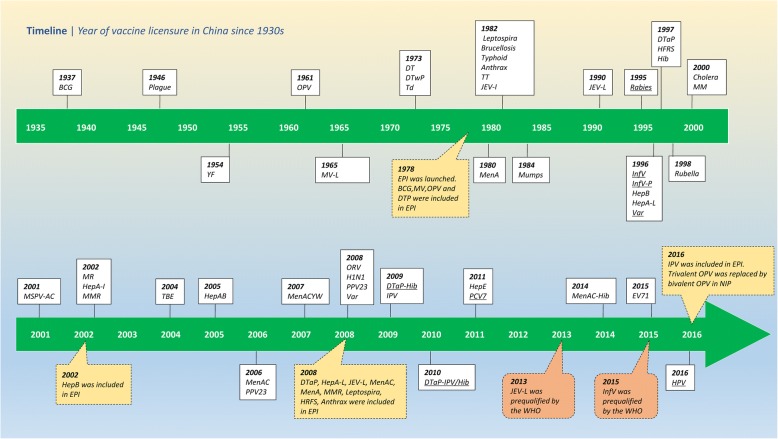
Timeline of initial vaccine licensure and inclusion in the immunization program since the 1930s. The yellow frames showed the NIP and EPI events, the orange frames showed the year of WHO prequalification. Underlined vaccines are imported vaccines

In China, 30 diseases are preventable by 50 vaccines (information on vaccine preventable diseases, vaccine categories, and years of EPI introduction are provided in Additional file 1: Figure S1) in 2007–2016. Figure 1 shows the vaccine licensure timeline since 1930. Seventeen vaccines were licensed between 1930 and 1990; no vaccines were licensed in 1990–1994; and 33 vaccines were licensed between 1995 and 2016. China developed novel vaccines including HepA-L, JEV-L, hepatitis E (HepE), and enterovirus 71 (EV71) vaccines. China developed several vaccines using different virus strains than are in common use globally, including MV (Shanghai-191 strain), oral rotavirus vaccine (ORV) (LLR-85 strain), rubella (BRD-2 strain), mumps (S79, developed from the Jeryl Lynn strain), Sabin-strain inactivated poliovirus vaccine (Sabin IPV), and HepA-L (H2). China Food and Drug Administration (CFDA) and the National Health and Family Planning Commission (NHFPC) have passed WHO assessments in 2011 and 2014, indicating that China’s vaccine regulation is at international/WHO standards. In 2013 and 2015, JEV-L and InfV were prequalified by WHO [13] (Fig. 1).

Although China produces almost all vaccines that are in common use globally, [2] compared with the US, the UK, and European countries, fewer vaccines are available in China. Meningococcal B (MenB) vaccine and adenovirus (Adenovirus) vaccine are not available in China (Additional file 1: Table S2). Human papillomavirus vaccine (HPV) was licensed in July 2016, ten years after licensure in some high-income countries. Multi-component vaccines such as MMR-Varicella (MMRV), HepB-Haemophilus influenzae type b conjugate vaccine (Hib), Typhoid-HepA, DTaP-IPV/HepB, and DTaP-Hib/Hep/IPV are not available in China.

### Expanded program of immunization

**Fig. 2:**
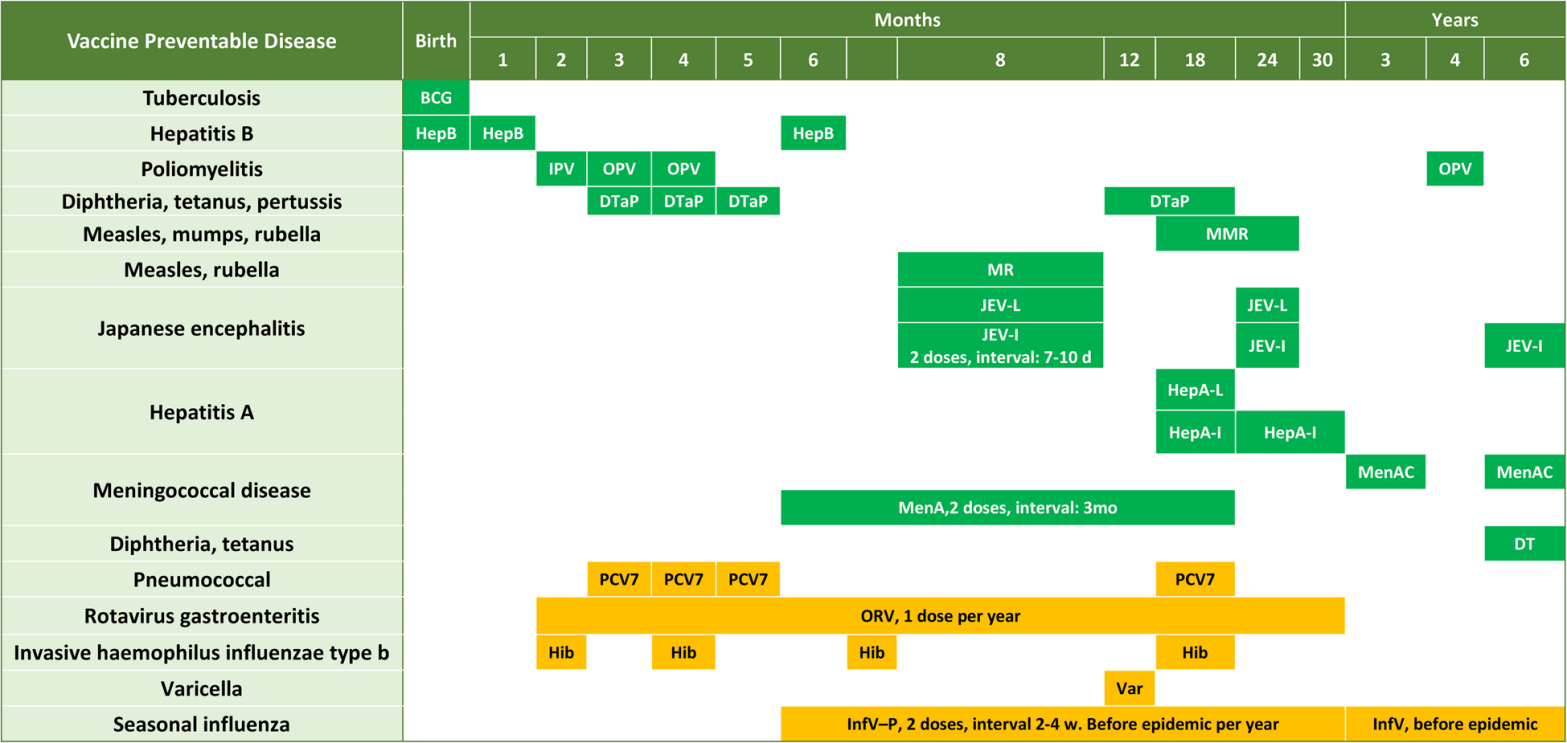
EPI and private-sector vaccination schedule from birth to 7 years old in China. EPI vaccines are shown in green. Vaccines shown in gold are private-sector vaccines that are recommended by WHO for inclusion in all national programs

In the 1950s, only smallpox and BCG vaccines were used universally in China, while diphtheria, JEV, Cholera, and Plague, and TT vaccines were used for epidemic control. China’s Expanded Program on Immunization was launched in 1978 with four vaccines: BCG, OPV, Measles vaccine, and DTwP. In the intervening 40 years, the list of EPI (Category 1) vaccines was increased 3 times: in 2001–2002, when HepB was included; in 2007–2008, when DTaP replaced DTwP and rubella, mumps, meningococcal polysaccharide, JEV-L, and HepA-L were included; and in 2016, when IPV was included. (Fig. 2).

Nineteen different EPI vaccines existed in 2007, decreasing to 13 in 2015 through substitution with combination vaccines. Compared with high-income countries (HICs) such as the UK and the US, China’s EPI system used more monovalent vaccines and lacked large combination vaccines like DTap-HepB/IPV and DTap-Hib/IPV (Additional file 1: Figure S1).

**Fig. 3:**
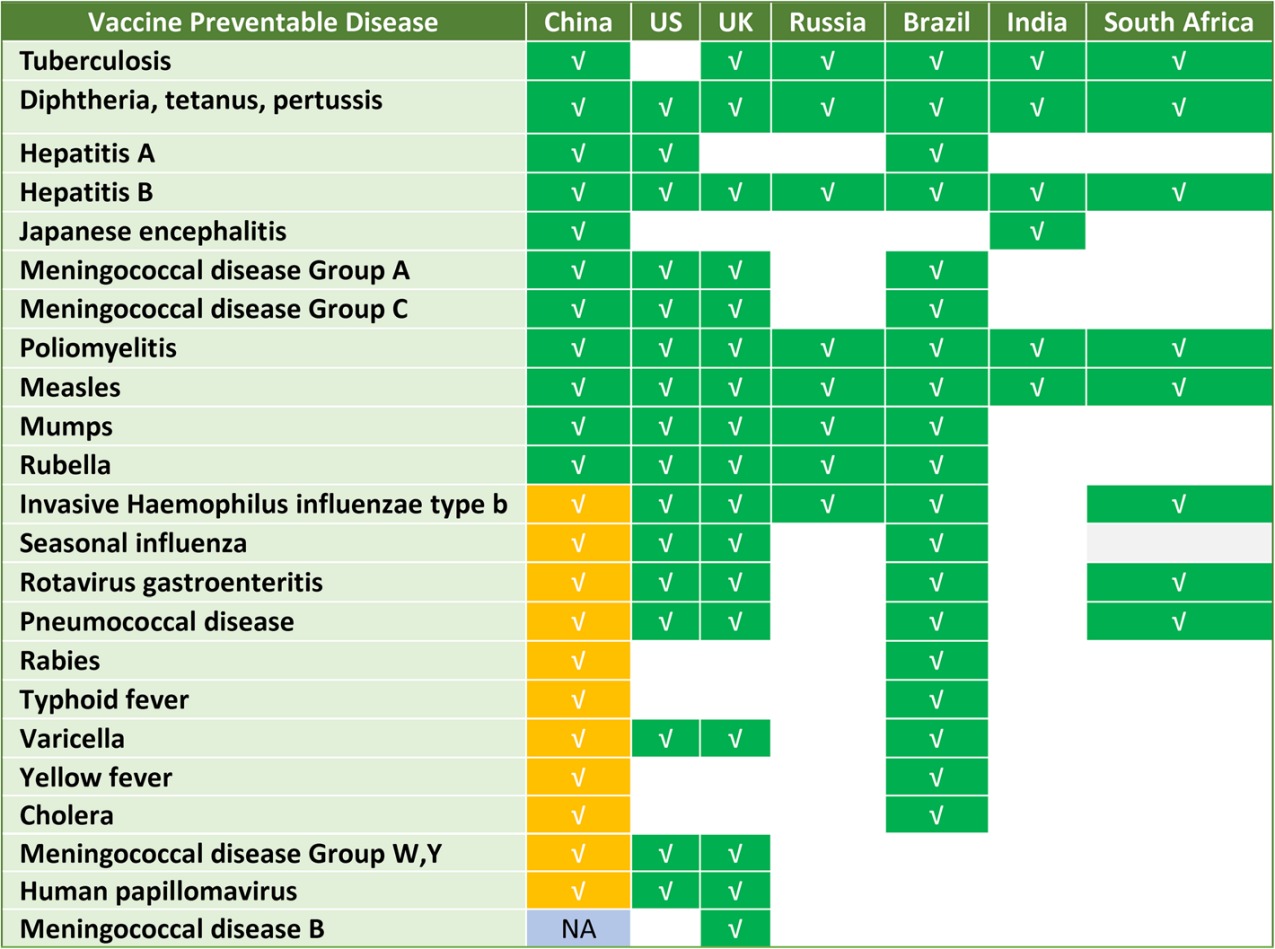
Vaccine Preventable Diseases (VPDs) for national immunization program vaccines in China, US, UK, Russia, Brazil, India and South Africa. VPDs indicated with green are EPI vaccines in the respective countries. Gold indicates diseases prevented by national programs in the UK, US, Russia, or Brazil’s NIP but whose vaccines are not included in China’s EPI system. Light blue indicates that the VPDs for which there is no licensed vaccines in China

Figure 3 depicts the national immunization program vaccines in high-income counties such as US and UK, middle income countries such as Russia, Brazil, India and South Africa. The National Immunization Program in China had more VPDs preventable by EPI vaccines than Russia, India and South Africa, but fewer VPDs preventable than the national programs in the US, UK and Brazil. Hib vaccine, pneumococcal conjugate vaccine (PCV) and rotavirus vaccine have been included in the US, UK, Brazil and South Africa’ national immunization programs, while China’s EPI has included none of these vaccines (Fig. 3). Hib vaccine, PCV13, oral rotavirus vaccine (ORV), varicella vaccine (Var), and seasonal influenza vaccine (InfV) were available only as Category 2 vaccines in China, while meningococcal group B (MenB) vaccine was not available in China.

### Categorization

With the increase number of vaccine available, the Chinese government implemented the 2-category administration of vaccines. In 2005, the Chinese government updated the legal framework for vaccines, *“Vaccine Distribution and Vaccination Regulations”* [14]. The law classified vaccines into two categories, 1 and 2. Category 1 includes all national-level EPI vaccines, supplemental vaccines paid for by lower-level governments, and emergency-use vaccines paid for by government. Category 1 vaccines are managed by government, and are provided free of charge to all children through 14 years of age. Timely immunization with Category 1 vaccines is considered a societal duty, although not a mandate as there are no punishments associated with noncompliance. Provincial CDCs procure vaccine from manufacturers and report demand and supply information to the central government. Vaccine manufacturers can only provide Category 1 vaccines to provincial or other CDCs in accordance with procurement contracts.

Category 2 (private-sector) vaccines are considered non-obligatory and must be paid for by the parent or person vaccinated. Rabies vaccine is a Category 2 vaccine even though its use in post-exposure prophylaxis following a bite from a rabid animal cannot be considered non-obligatory. Prior to April 2016, manufacturers were allowed to sell Category 2 vaccines to wholesalers, county or district CDCs, and vaccination clinics and other healthcare providers. In 2016, the Chinese government revised the legal framework for vaccines and immunization (“Vaccine Distribution and Vaccination Regulations”) in partial response to an illegal Category 2 vaccine sales ring based in Shandong province in 2016 [15]. The updated law specified that all Category 2 vaccines must be procured using government procurement platforms, [16] essentially requiring Category 2 vaccines to be managed by the system that manages Category 1 vaccines. The updated law bans vaccine sales through the Internet and wholesalers, instead directing all distribution through CDCs. In January 2017, the Chinese State Council again updated the Vaccine Circulation and Immunization regulation. The update requested establishment of a national advisory committee of experts to make recommendations for inclusion of vaccines into the EPI system based on vaccine safety, effectiveness, cost-effectiveness, and production capacity and to update recommendations on existing EPI vaccines. The update also encouraged new vaccine innovation through national science projects.

### Vaccine supply

From 2007 to 2015, 55 vaccines were licensed in China; 48 (78%) were manufactured domestically. The number of manufacturers for each vaccine varies from 1 (for TBE vaccine) to 21 (for InfV) (Additional file 1: Table S1). Most of the vaccines manufactured in China were used domestically. Three vaccines (JEV-L made by Chengdu Institute of Biological Products; InfV, made by Hualan Biological; and inactivated hepatitis A vaccine, made by Sinovac Biotech Co. Ltd.) have been prequalified by WHO, [1, 16] enabling procurement by UNICEF and Gavi for use in other countries.

**Fig. 4:**
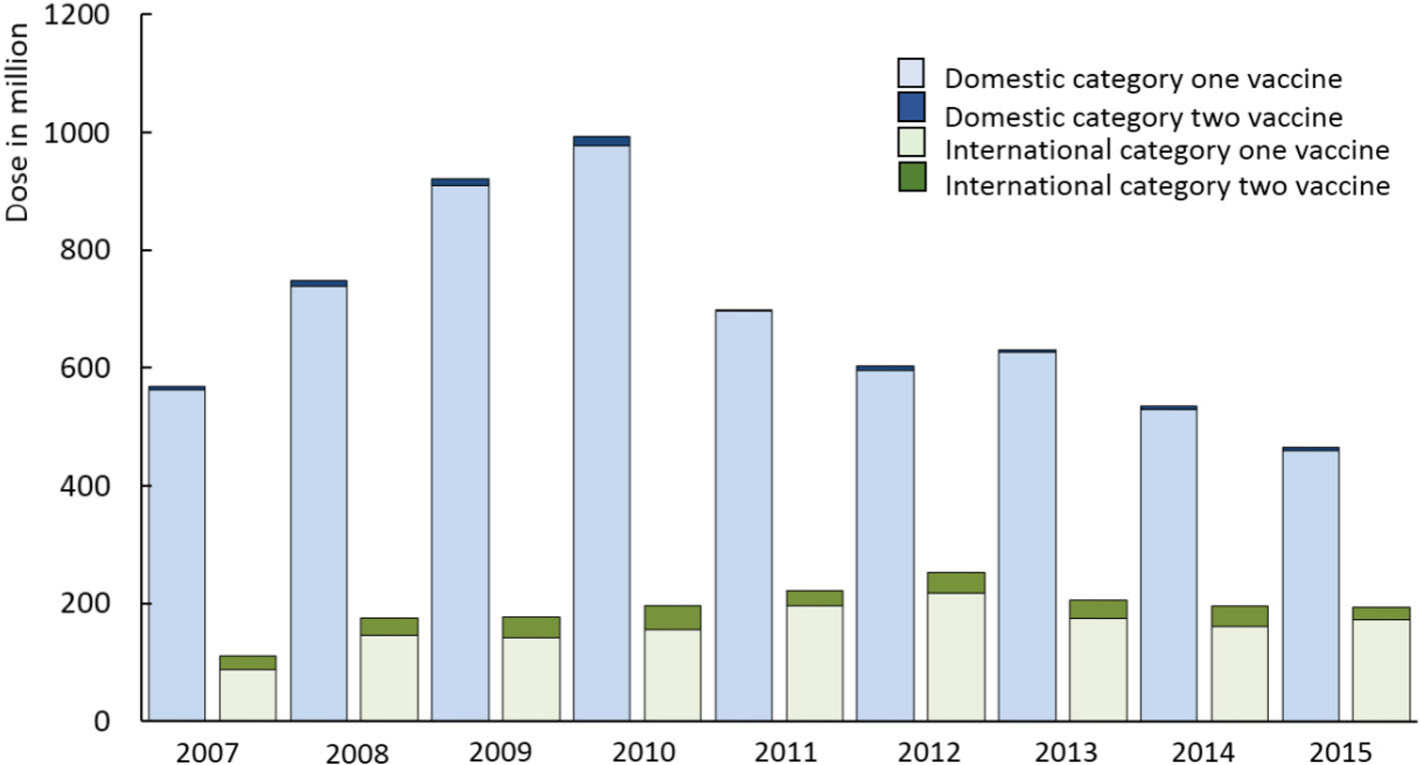
Supply volumes of domestic and international Category 1 (EPI) and 2 (private sector) vaccines from 2007 to 2015. The fluctuation of supply of Category 1 vaccines was largely due to vaccination campaigns. The supply of H1N1 reached 70 million doses in 2010. The supply of HepB and MV increased by 83% and 202% in 2010 for supplementary immunization activities

Figure 4 shows the number of vaccine doses distributed by year, broken down by category and location of production (domestic or international). Between 2007 and 2015, the supply volume varied from 666 million doses to 1.19 billion doses, with Category 1 vaccines accounting for the majority of vaccines supplied. Virtually all Category 1 vaccines were produced domestically, as were approximately 80% to 90% of Category 2 vaccines. Variation in the supply of Category 1 vaccine reflects national campaigns with H1N1 vaccine, HepB vaccine, and monovalent MV-L vaccine [9, 17, 18].

### Price

**Fig. 5:**
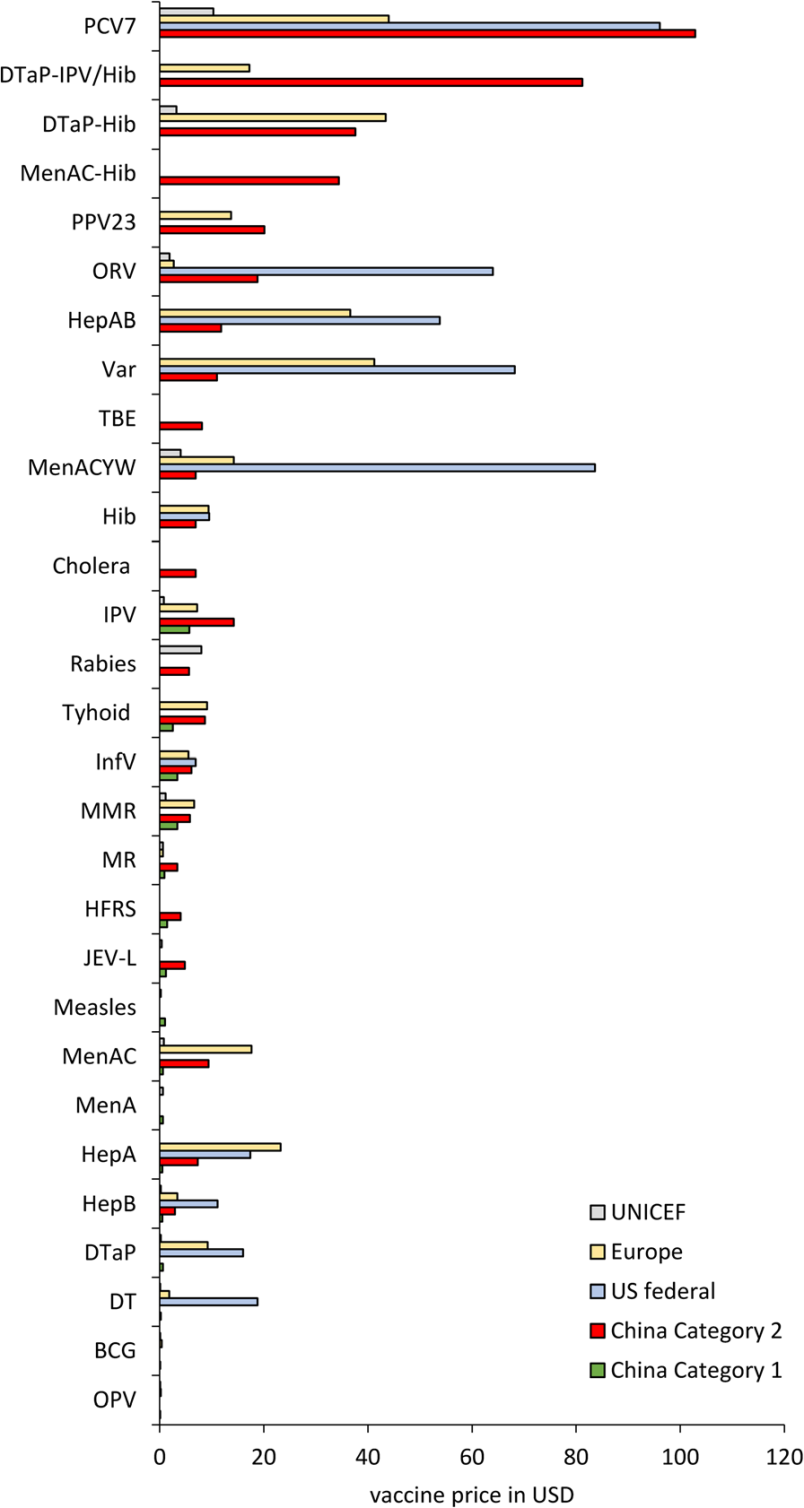
Comparison of prices of vaccines among China, US, European countries and UNICEF (The exchange rate for US dollars - Chinese Yuan was 6.2)

In China, both Category 1 and Category 2 vaccines are procured by government using a centralized platform. Category 1 vaccines are provided free of charge, while Category 2 vaccines are paid for out-of-pocket by the family. Figure 5 shows vaccine procurement prices in China, US, European countries, and UNICEF. The price range for Category 1 vaccines was $0.1–5.7 USD, and the price range for Category 2 vaccines was $2.4–102.9 USD. Procurement prices of EPI-substitute vaccines (Category 2 vaccines against diseases preventable by Category 1 vaccines) were higher than for their EPI equivalents.

Category 1 vaccine prices were lower than for the same vaccines in the US and European countries, but were similar to UNICEF prices. Prices of domestically-produced Category 2 vaccines, such as seasonal influenza, Hib, Var, and ORV, were lower than prices of similar vaccines in the US, Europe, and UNICEF. PCV7 and DTaP-IPV/Hib were higher in price than similar vaccines in the US, Europe and UNICEF (Fig. 5).

## Discussion

Despite significant achievements in vaccine development and production, and the attainment of important goals for polio eradication, hepatitis B control, and tetanus elimination in the past two decades, China’s EPI system protects children from fewer diseases than many high-income and middle-income countries. Notable is the lack Hib, ORV, PCV, influenza and HPV vaccines in China’s EPI system - vaccines that are recommended by WHO for all countries’ national immunization programs but are available only as private-sector vaccines in China.

China’s immunization program has made very good use of the EPI vaccines. Polio was eradicated by 2000; [19] the prevalence of chronic hepatitis B infection has been reduced by 97% among young children compared with the pre-vaccine era; [17] the annual incidence of measles has been reduced by more than 99% [18]. Strategic use of effective vaccines made these achievements possible, despite the challenges of a large population and a vast territory with many densely-populated areas. There are several experiences that could be helpful in low-income countries: 1) China has a large domestic manufacturing base capable of producing large quantity of vaccines at low prices, which has enabled all eligible children to be vaccinated free of charge; 2) the Chinese government has encouraged through policy development expansion of the number of diseases preventable by vaccines; and 3) stringent requirements ensuring access to vaccines through thousands of CDCs and over 200,000 vaccination clinics have ensured high coverage of EPI vaccines. We believe that China’s immunization program experience can help other countries achieve similar results with effective, inexpensive vaccines.

In industrialized countries like the US and the UK, almost all vaccines are regarded as “public goods,” ensuring their availability for all children [20, 21]. However, some vaccines that WHO recommends for all national immunization programs are Category 2 vaccines in China, which require out-of-pocket payment by parents or persons vaccinated. Category 2 vaccines are associated with lower coverage levels and with coverage that varies by wealth of province. For example, coverage with ORV is 23.7%, and coverage with Hib vaccine is 45.3%. China ranks 7th highest in the world for VPD burden, indicating that more complete implementation of underutilized vaccines can contribute to a healthier childhood population [22].

The fluctuation of vaccine supply in 2007–2015 shows that the vaccine demand depends on the birth cohort and the government-oriented catch up campaigns. In 2015, the amendment of “Population and Family Planning Law of” was changed China’s one-child policy to a two-child policy. This change will lead to an increase in the birth cohort size and to an increase in vaccine demand.

The prices of China’s Category 1 vaccines were much lower than high-income country and some UNICEF vaccine prices. Low vaccine prices may provide manufacturers with less incentive to invest in production improvements and new vaccine development, which may be a partial explanation for the lack of domestic production of large combination vaccines. From a short- to medium-term public health perspective, lower-cost vaccines are preferable because more children can be vaccinated for a given budget [23]. Low pricing can be achieved in part through large demands in an economy of scale [24]. Prices for Category 2 vaccines may be high because their demand volumes are not large enough to achieve an economy of scale. Manufacturers of Category 2 vaccines profit from high prices, which provides an incentive to stay in the market and fund innovations in research and development of novel vaccines. Finding a balance between manufacturers’ profit needs and public health’s needs is challenging. The 2-category system in China is one approach to finding a balance, with traditional vaccines in the program and newer vaccines available in China, but not provided by the program.

Our study has limitations and strengths. Limitations include (1) that procurement prices can vary by province, and we were not able to capture that variation in the study, and (2) that CFDA lot-release program data were not available prior to 2007 [1]. A strength is that we used official government-reported vaccine licensure information, dose amounts, and pricing. For example, CFDA’s lot-release program was the source of information on the number of doses available in China, and government procurement offices were the source for price information.

## Conclusions

We believe that our study supports two recommendations. First, China’s government should induce domestic manufacturers to develop and license vaccines recommended by WHO for inclusion in all national immunization programs. Lack of domestic manufacturers for vaccines such as PCV and HPV vaccine delay introduction into the EPI system. Second, Chinese vaccine manufacturers should be encouraged to participate in the WHO vaccine prequalification program. Vaccines such as bivalent OPV, Sabin IPV, MR, HepE, and EV71 can contribute to the WHO prequalification program and help prevent VPDs in other countries.

## Additional file

Additional file 1: **Table S1.** Vaccine supplied in China, 2007–2016; The table contains the vaccine preventable diseases, name of vaccine, acronyms, category, year of license, year of EPI, number of domestic and international manufacturers. **Table S2.** The vaccines not supplied in China but in UK and US; The table contains the vacciens supplied in UK and USA but not supplied in China. **Figure S1.** The national immunization program vaccines in China, UK, US, Russia, Brazil, India and South Africa. The figure contains vaccines integrated in EPI in China, UK, US, Russia, Brazil, India and South Africa in Green and vaccines not included in EPI in China in Gold. (DOCX 40 kb)

## Abbreviations

Anthrax: Anthrax vaccine
BCG: Bacillus Calmette-Guerin vaccine
Brucellosis: Brucellosis vaccine
Cholera: B recombinant subunit /cholera vaccine
DT: Diphtheria and tetanus combined vaccine
DTaP -IPV/Hib: Diphtheria-tetanus-pertussis whole cell vaccine, inactivated poliovirus vaccine and Haemophilus influenza type b conjugate vaccine
DTaP: Diphtheria-tetanus-pertussis acellular vaccine
DTaP-Hib: Diphtheria-tetanus-acellular pertussis combined vaccine and Haemophilus influenza type b conjugate vaccine
DTwP: Diphtheria-tetanus-pertussis whole cell vaccine
EPI: Expanded program of immunization
EV71: Enterovirus vaccine, inactivated
H1N1: Pandemic H1N1 vaccine
HepAB: Hepatitis A and hepatitis B vaccines
HepA-I: Hepatitis A, inactivated
HepA-L: Hepatitis A, live attenuated
HepB: Hepatitis B vaccine, recombinant
HepE: Hepatitis E vaccine
HFRS: Hemorrhagic fever with renal syndrome vaccine, inactivated
Hib: Haemophilus influenza type b conjugate vaccine
HPV: Human papillomavirus vaccine
InfV: Influenza vaccine
InfV-P: Influenza vaccine, Pediatric
IPV: Inactivated poliovirus vaccine
JEV-I: Japanese encephalitis vaccine, inactivated
JEV-L: Japanese encephalitis vaccine, live attenuated
Leptospira: Leptospirosis vaccine
MenA: Group A meningococcal polysaccharide vaccine
MenAC: Group A, C meningococcal polysaccharide conjugant vaccine
MenAC-Hib: Group A,C meningococcal polysaccharide conjugant vaccine and Haemophilus influenzae type b conjugate vaccine
MenACYW: Group ACYW 135 meningococcal polysaccharide vaccine
MM: Measles, mumps combined vaccine, live attenuated
MMR: Measles-mumps-rubella vaccine, live attenuated
MPSV-AC: Group A,C meningococcal polysaccharide vaccine
MR: Measles-rubella vaccine, live attenuated
Mumps: Mumps vaccine, live attenuated
MV-L: Measles vaccine, live attenuated
NIP: national immunization program
OPV: Oral Poliovirus vaccine, live attenuated
ORV: Oral rotavirus vaccine
PCV13: Pneumococcal conjugate vaccine (13-valent)
PCV7: Pneumococcal conjugate vaccine (7-valent)
Plague: Plague vaccine
PPV23: Pneumococcal polysaccharide vaccine (23-valent)
Rabies: Rabies vaccine
Rubella: Rubella, live attenuated
TBE: Tick-borne encephalitis vaccine, inactivated
Td: Diphtheria and tetanus combined vaccine, adult
Tdap: Diphtheria-tetanus-pertussis acellular vaccine, adult
TT: Tetanus toxoid vaccine
Typhoid: Typhoid Vi polysaccharide vaccine
Var: Varicella vaccine
YF: Yellow fever vaccine
