# Supplementary material for: The landscape of vaccines in China: history, classification, supply, and price
Source: BMC Infect Dis. 2018 Oct 4;18:502. doi: 10.1186/s12879-018-3422-0 (PMC6172750; doi:10.1186/s12879-018-3422-0)
Supplement: Supplementary file 1 — Table S1. Vaccine supplied in China, 2007–2016; The table contains the vaccine preventable diseases, name of vaccine, acronyms, category, year of license, year of EPI, number of domestic and international manufacturers. Table S2. The vaccines not supplied in China but in UK and US; The table contains the vacciens supplied in UK and USA but not supplied in China. Figure S1. The national immunization program vaccines in China, UK, US, Russia, Brazil, India and South Africa. The figure contains vaccines integrated in EPI in China, UK, US, Russia, Brazil, India and South Africa in Green and vaccines not included in EPI in China in Gold. (DOCX 40 kb) [file 12879_2018_3422_MOESM1_ESM.docx]

**Additional file 1: Table S1. Vaccine supplied in China, 2007-2016**

| No. | Vaccine Preventable Disease | Name of vaccine | Acronyms | Category | Yr. Licensed | Year EPI | Dom* | Intl* |
| --- | --- | --- | --- | --- | --- | --- | --- | --- |
| 1 | Pneumococcal Pneumonia | Pneumococcal conjugate vaccine (7-valent) | PCV7 | 2 | 2011 | --- | 0 | 1 |
|  |  | Pneumococcal conjugate vaccine (13-valent) | PCV13 | 2 | 2016 | --- | 0 | 1 |
|  |  | Pneumococcal polysaccharide vaccine (23-valent) | PPV23 | 2 | 2008 | --- | 1 | 2 |
| 2 | Pneumonia and Meningitis | Haemophilus influenza type b conjugate vaccine | Hib | 2 | 1997 | --- | 4 | 4 |
| 3 | Rabies | Rabies vaccine | Rabies | 2 | 1995 | --- | 17 | 1 |
| 4 | Varicella | Varicella vaccine | Var | 2 | 1996 | --- | 5 | 1 |
| 5 | Diarrhea | Oral rotavirus vaccine | ORV | 2 | 1998 | --- | 1 | 0 |
| 6 | Brucellosis | Brucellosis vaccine | Brucellosis | 2 | 1982 | --- | 1 | 0 |
| 7 | Seasonal influenza | Influenza vaccine, Pediatric | InfV-P | 2 | 1996 | --- | 10 | 3 |
|  |  | Influenza vaccine | InfV | 2 | 1996 | --- | 14 | 7 |
| 8 | Pestis | Plague vaccine | Plague | 2 | 1946 | --- | 1 | 0 |
| 9 | Tick-bone Encephalitis | Tick-borne encephalitis vaccine, inactivated | TBE | 2 | 2004 | --- | 1 | 0 |
| 10 | Typhoid fever | Typhoid Vi polysaccharide vaccine | Typhoid | 2 | 1982 | --- | 4 | 0 |
| 11 | Cholera | B recombinant subunit /cholera vaccine | Cholera | 2 | 2000 | --- | 1 | 0 |
| 12 | Yellow fever | Yellow fever vaccine | YF | 2 | 1954 | --- | 1 | 0 |
| 13 | Polio | Inactivated poliovirus vaccine | IPV | 2 | 2009 | --- | 1 | 1 |
|  |  | Oral Poliovirus vaccine, live attenuated | OPV | 1 | 1961 | 1978 | 2 | 0 |
| 14 | Meningococcal meningitis | Group ACYW 135 meningococcal polysaccharide vaccine | MenACYW | 2 | 2007 | --- | 6 | 0 |
|  |  | Group A meningococcal polysaccharide vaccine | MenA | 1 | 1980 | 2007 | 3 | 0 |
|  |  | Group A,C meningococcal polysaccharide vaccine | MPSV-AC | 1 | 2001 | 2007 | 6 | 1 |
|  |  | Group A,C meningococcal polysaccharide conjugant vaccine | MenAC | 1 | 2006 | 2007 | 3 | 0 |
|  |  | Group A,C meningococcal polysaccharide conjugant vaccine and Haemophilus influenzae type b conjugate vaccine | MenAC-Hib | 2 | 2014 | --- | 1 | 0 |
| 15 | Hepatitis | Hepatitis A, live attenuated | HepA-L | 1 | 1996 | 2007 | 3 | 5 |
|  |  | Hepatitis A, inactivated | HepA-I | 2 | 2002 | --- | 4 | 0 |
|  |  | Hepatitis B vaccine, recombinant | HepB | 1 | 1996 | 2002 | 8 | 2 |
|  |  | Hepatitis A and hepatitis B vaccines | HepAB | 2 | 2005 | --- | 1 | 1 |
|  |  | Hepatitis E vaccine | HepE | 2 | 2011 | --- | 1 | 0 |
| 16 | Tuberculosis | Bacillus Calmette-Guerin vaccine | BCG | 1 | 1937 | 1954 | 2 | 0 |
| 17 | Diphtheria, Tetanus, Pertussis | Diphtheria-tetanus-pertussis whole cell vaccine | DTwP | 1 | 1973 | 1978 | 3 | 0 |
|  |  | Diphtheria-tetanus-pertussis acellular vaccine | DTaP | 1 | 1997 | 2007 | 5 | 1 |
|  |  | Diphtheria-tetanus-pertussis acellular vaccine, adult | Tdap | 2 | 1997 | --- | 0 | 1 |
|  |  | Diphtheria and tetanus combined vaccine | DT | 1 | 1973 | 1978 | 3 | 0 |
|  |  | Diphtheria and tetanus combined vaccine, adult | Td | 2 | 1973 | --- | 2 | 0 |
|  |  | Tetanus toxoid vaccine | TT | 1 | 1982 | 1978 | 4 | 0 |
|  |  | Diphtheria-tetanus-acellular pertussis combined vaccine and Haemophilus influenza type b conjugate vaccine | DTaP-Hib | 2 | 2009 | --- | 1 | 2 |
|  |  | Diphtheria-tetanus-pertussis whole cell vaccine, inactivated poliovirus vaccine and Haemophilus influenza type b conjugate vaccine | DTaP -IPV/Hib | 2 | 2010 | --- | 0 | 1 |
| 18 | Japanese encephalitis | Japanese encephalitis vaccine, live attenuated | JEV-L | 1 | 1990 | 2007 | 3 | 0 |
|  |  | Japanese encephalitis vaccine, inactivated | JEV-I | 1 | 1982 | --- | 4 | 0 |
| 19 | Leptospirosis | Leptospirosis vaccine | Leptospira | 1 | 1982 | 2007 | 2 | 0 |
| 20 | Measles,Mumps,Rubella | Measles-rubella vaccine, live attenuated | MR | 1 | 2002 | 2007 | 2 | 0 |
|  |  | Measles, mumps combined vaccine, live attenuated | MM | 1 | 2000 | 2007 | 2 | 0 |
|  |  | Measles-mumps-rubella vaccine, live attenuated | MMR | 1 | 2002 | 2007 | 2 | 2 |
|  |  | Measles vaccine, live attenuated | MV-L | 1 | 1965 | 1978 | 5 | 0 |
|  |  | Mumps vaccine, live attenuated | Mumps | 1 | 1984 | 2007 | 5 | 0 |
|  |  | Rubella, live attenuated | Rubella | 1 | 1998 | 2007 | 2 | 0 |
| 21 | Anthrax | Anthrax vaccine | Anthrax | 1 | 1982 | 2007 | 1 | 0 |
| 22 | Hemorrhagic fever with renal syndrome | Hemorrhagic fever with renal syndrome vaccine, inactivated | HFRS | 1 | 1997 | 2007 | 4 | 0 |
| 23 | Pandemic influenza | Pandemic H1N1 vaccine | H1N1 | 1 | 2008 | --- | 10 | 0 |
| 24 | Enterovirus 71 | Enterovirus vaccine, inactivated | EV71 | 2 | 2015 | --- | 2 | 0 |
| 25 | Human papillomavirus | Human papillomavirus vaccine(16, 18) | HPV | 2 | 2016 | --- | 0 | 1 |

*Dom refers to the number of domestic manufacturers; Intl refers to the number of international manufacturers.

**Additional file 1: Table S2.** The vaccines not supplied in China but in UK and US

| Vaccine preventable diseases | UK | US |
| --- | --- | --- |
| Meningococcal Group B | MenB | MenB |
| Herpes Zoster, Shingles | HZV | HZV |
| Adenovirus |  | Adenovirus |

| Vaccine Preventable Disease | China | US | UK | Russia | Brazil | India | South Africa |
| --- | --- | --- | --- | --- | --- | --- | --- |
| Tuberculosis | BCG |  | BCG | BCG | BCG | BCG | BCG |
| Diphtheria, tetanus, pertussis | DT  DTaP | DTaP-Hib/IPV  DTaP-HepB/IPV  DTaP-IPV  Tdap | DTaP-Hib/IPV  DTaP-IPV  Td-IPV | DT, Td  Dip  DTwP  DTwP-Hep | DT, Td  DTaP  DTwP  DTwP-Hib/Hep | DTwP  DTwP-Hib/HepB  TT | DTaP-Hib/IPV  TT, Td |
| Hepatitis A | HepA | HepA |  |  | HepA |  |  |
| Hepatitis B | HepB | DTaP-HepB/IPV  HepB | HepB | HepB | HepB | HepB | HepB |
| Japanese Encephalitis | JE |  |  |  |  | JE |  |
| Meningococcal disease Group A | MenA | MenACYW | MenACYW |  | MenAC |  |  |
| Meningococcal disease Group C | MenAC | MenACYW | Hib-MenC  MenC |  | MenAC |  |  |
| Poliomyelitis | IPV  OPV | IPV  DTaP-Hib/IPV  DTaP-HepB/IPV  DTaP-IPV | DTaP-Hib/IPV  DTaP-IPV | OPV | OPV  IPV | OPV | OPV,  DTaP-Hib/IPV |
| Measles | MMR  MR | MMR | MMR | MMR, MM,  Mumps, Rubella | MMR | Measles | Measles |
| Mumps |  |  |  |  |  |  |  |
| Rubella |  |  |  |  |  |  |  |
| Invasive Haemophilus influenzae type b | Hib | Hib | Hib-MenC  DTaP-Hib/IPV  DTaP-IPV | Hib | DTwP-Hib/Hep  Hib |  | DTaP-Hib/IPV |
| Seasonal influenza | InfV | InfV | InfV |  | InfV |  |  |
| Rotavirus gastroenteritis | ORV | ORV | ORV |  | ORV |  | ORV |
| Pneumococcal disease | PPV23  PCV13  PCV7 | PPV23  PCV10, PCV13 | PPV23  PCV10,PCV13 |  | PPV  PCV |  | PCV |
| Rabies | Rabies |  |  |  | Rabies |  |  |
| Typhoid fever | Typhoid |  |  |  | Typhoid |  |  |
| Varicella | Var | Var | Var |  | Var |  |  |
| Yellow fever | YF |  |  |  | YF |  |  |
| Cholera | Cholera |  |  |  | Cholera |  |  |
| Meningococcal disease Group W,Y | MenACYW | MenACYW | MenACYW |  |  |  |  |
| Human papilloma virus | HPV | HPV | HPV |  |  |  |  |
| Meningococcal disease B | NA |  | MenB |  |  |  |  |

**Additional file 1: Figure S1. The national immunization program vaccines in China, UK, US, Russia, Brazil, India and South Africa**

Gold indicates diseases prevented by national programs in the UK, US, Russia, or Brazil’s NIP but whose vaccines are not included in China’s EPI system. Light blue indicates that the VPDs for which there is no licensed vaccines in China.
